# A Novel Insight into the Oxidoreductase Activity of *Helicobacter pylori* HP0231 Protein

**DOI:** 10.1371/journal.pone.0046563

**Published:** 2012-10-03

**Authors:** Paula Roszczenko, Katarzyna A. Radomska, Ewa Wywial, Jean-Francois Collet, Elzbieta K. Jagusztyn-Krynicka

**Affiliations:** 1 Department of Bacterial Genetics, Institute of Microbiology, the University of Warsaw, Warsaw, Poland; 2 College of Inter-Faculty Individual Studies in Mathematics and Natural Sciences, the University of Warsaw, Warsaw, Poland; 3 Laboratory of Bioinformatics and Protein Engineering, International Institute of Molecular and Cell Biology, Warsaw, Poland; 4 WELBIO (Walloon Excellence in Life Sciences and Biotechnology), Université Catholique de Louvain, Brussels, Belgium; 5 de Duve Institute, Université Catholique de Louvain, Brussels, Belgium; 6 Brussels Center for Redox Biology, Brussels, Belgium; Veterans Affairs Medical Center (111D), United States of America

## Abstract

**Background:**

The formation of a disulfide bond between two cysteine residues stabilizes protein structure. Although we now have a good understanding of the *Escherichia coli* disulfide formation system, the machineries at work in other bacteria, including pathogens, are poorly characterized. Thus, the objective of this work was to improve our understanding of the disulfide formation machinery of *Helicobacter pylori*, a leading cause of ulcers and a risk factor for stomach cancer worldwide.

**Methods and Results:**

The protein HP0231 from *H. pylori*, a structural counterpart of *E. coli* DsbG, is the focus of this research. Its function was clarified by using a combination of biochemical, microbiological and genetic approaches. In particular, we determined the biochemical properties of HP0231 as well as its redox state in *H. pylori* cells.

**Conclusion:**

Altogether our results show that HP0231 is an oxidoreductase that catalyzes disulfide bond formation in the periplasm. We propose to call it HpDsbA.

## Introduction

Disulfide bond formation is a post-translational modification, which stabilizes the structure of many extracytoplasmic proteins. In bacteria, disulfides are introduced into proteins secreted to the periplasm by proteins from the Dsb family. In *Escherichia coli* (Ec), the disulfide bond formation machinery involves two complementary pathways: the oxidation pathway, with EcDsbA and EcDsbB, and the isomerization/reduction pathway, with EcDsbC and EcDsbD [1], [2]. The first pathway is responsible for the formation of disulfide bonds in newly synthesized proteins, while the second catalyzes the rearrangement of improperly introduced disulfides [3].

EcDsbA is a 21 kDa monomeric protein from the thioredoxin family, which donates the disulfide bond present in its active site to reduced protein substrates. This reaction depends on the presence of two highly conserved motifs: a CXXC (CPHC) active site motif and the so-called *cis*-proline loop [4]. Both motifs are distant in linear sequence but close in the three-dimensional structure. EcDsbA is maintained in an oxidized form *in vivo* due to the action of EcDsbB, a 20 kDa membrane protein that generates disulfides *de novo* from quinone reduction. Thus, disulfide bond formation is connected to the electron transport chain.

EcDsbA does not have a rearranging activity and can introduce non-native disulfides into proteins with multiple cysteine residues. In *E. coli*, the periplasmic protein disulfide isomerase EcDsbC is responsible for the rearrangement of those incorrect disulfides. EcDsbC is a 23.3 kDa protein that folds into a V-shaped homo-dimer [5]. Each monomer of DsbC consists of two domains: a C-terminal catalytic domain with a thioredoxin fold and a N-terminal dimerization domain that also plays a role in substrate binding.

In *E. coli*, the periplasm contains an additional oxidoreductase, EcDsbG, which is a structural homologue of EcDsbC [6]. EcDsbG has recently been shown to control the levels of cysteine sulfenylation in the periplasm [7].

EcDsbC and EcDsbG both have catalytic CXXC motifs that are kept in the reduced state by the integral membrane protein EcDsbD. EcDsbD, which catalyzes the transfer of electrons from the cytoplasmic thioredoxin system to the periplasm, consists of eight transmembrane segments (β domain), a N-terminal (α domain) and a C-terminal domain (γ domain). Both the N- and C-terminal domains are present in the periplasm [8].

In contrast to the *E. coli* system which has been extensively studied, the *H. pylori* disulfide bond formation system has been poorly characterized although this system plays an important role in the colonization process [9]. Previous research has revealed that the *H. pylori* genome encodes a homologue of EcDsbB (HpDsbI) [10] but no close homologue of EcDsbA. There are also two additional thioredoxin-like CXXC-containing periplasmic proteins, HP0231 and HP0377. These proteins share only 12% and 24% identity with EcDsbA, respectively, and their function is unknown [11].

*H. pylori* infection induces a strong inflammatory response in the stomach. This response is manifested by generation of large amounts of reactive oxygen species/reactive nitrogen species (ROS/RNS) produced by infiltrating host cells. The long-term exposure to ROS contributes to the development of cancerous gastric cells [12], [13]. Thus, the pathogen has evolved a defense system involving several antioxidant enzymes, such as superoxide dismutase, catalase or alkyl hyperoxide reductase [14]. Additionally, oxidoreductases, specifically disulfides reductases, play a major role in the antioxidant defense mechanism and in the maintenance of intracellular redox balance. Some of them can even act in the oxidative environment of the cell envelope [15]. Because the *H. pylori* is equipped with only one intracellular disulfide reducing system, the thioredoxin system, and lacks the glutaredoxin/glutathione system and because the *H. pylori* Dsb system is still poorly understood molecular characterization of the Dsb proteins may also help to expand our knowledge about the mechanism used by *H. pylori* to combat oxidative stress.

We decided to genetically and biochemically study HP0231, a protein which has been selected as a component of a subunit anti-*Helicobacter* vaccine [16], [17], to shed light on its function. To achieve this goal, we generated a *H. pylori hp0231* mutant and subsequently carried out its phenotypic characterization. We also examined whether HP0231 is able to complement an *E. coli dsbA* mutant and we biochemically characterized the protein. Moreover, we determined the redox state of HP0231 in wild-type and a *dsbI* mutant strain of *H. pylori*.

## Materials and Methods

### Ethics Statement

All studies involving animals were performed in accordance with ethical standards, after approval from the Local Ethic Committee No. 1 Warsaw, Poland 966/2009.

### Bacterial strains, primers, plasmids, media and growth conditions

Bacterial strains, plasmids and primers used in this study are listed in Tables 1 and 2. Two *H. pylori* strains (26695 and N6) were used in this study. Although the sequence of the *H. pylori* 26695 genome is completed [18], this strain is inconvenient for genetic manipulation. *H. pylori* N6, originally isolated from a patient with gastritis, is useful for complementation experiments and highly motile [19]. Both *H. pylori* strains were grown on blood agar base 2 (BA) plates (Merck) supplemented with 10% horse blood and an antibiotic mixture consisting of vancomycin (final concentration, 12.5 µg ml^−1^), polymyxin B (1.25 µg ml^−1^), trimethoprim (6.25 µg ml^−1^) and amphotericin B (2.5 µg ml^−1^) at 37°C under microaerobic conditions by using CampyGen (Oxoid). Liquid cultures of *H. pylori* were grown in Brain Heart Infusion (BHI) broth supplemented with 10% fetal bovine serum (FBS). For the selection of *H. pylori* mutated or complemented strains, kanamycin (25 µg ml^−1^) or/and chloramphenicol (10 µg ml^−1^) were added to the growth media.

**Table 1:** Bacterial strains and plasmids used in this study.

Name	Relevant characteristics	Source/Ref.
Strains: *Helicobacter pylori*		
26696	*H. pylori* wild-type	ATCC
N6	*H. pylori* wild-type	[19]
PR305	N6 *dsbI*::*aph*	This study
PR336	*dsbI* ^+^ *in trans* complementant of *dsbI*::*aph*	This study
PR378	N6 *hp0231*::*cat*	This study
PR397	*hp0231* ^+^ *in trans* complementant of *hp0231*::*cat*	This study
*Escherichia coli*		
TG1	*supE44 hsd*Δ *5 thi* Δ(*lac^−^ proAB*) F′ [*traD36 proAB^+^ lacI^q^ lacZ*ΔM15]	[21]
BL21	F^−^ *ompT hsdS_B_*(*r_B_^−^m_B_^−^*) *gal dcm lon*	Novagen
JCB816	MC1000 *phoR* λ102	[44]
JCB817	JCB816 *dsbA*::*kan1*	[44]
JCB818	JCB 816 *dsbA*::*kan1 dsbB*::*kan*	[44]
JFC383	JCB816 *dsbC*::*kan*	JFC collection
JFC571	JFC383 pHEL2	This study
JFC572	JFC383 pUWM500	This study
PR501	JCB 817 pHEL2	This study
PR503	JCB 817 pUWM500	This study
PR521	JCB 818 pHEL2	This study
PR522	JCB 818 pUWM500	This study
Plasmids:		
pET22b	Ap^r^, IPTG inducible	Novagen
pET28a	Km^r^, IPTG inducible	Novagen
pGEM-T Easy	Ap^r^; LacZα	Promega
pHEL2	Cm^r^ *E. coli/H. pylori* shuttle vector	[45]
pHEL3	Km^r^ *E. coli/H. pylori* shuttle vector	[45]
pRY109	Cm^r^	[41]
pUWM305	pBluescript II SK/*dsbI*::*aph*	[10]
pUWM336	*dsbI* ^+^ in pHEL2	[10]
pUWM378	pGEM-T Easy/*hp0231*::*cat*	This study
pUWM382	Ap^r^ *hp0231* ^+^ in pET22b	This study
pUWM589	*hp0231* ^+^ in pGEM-T Easy	This study
pUWM397	*hp0231* ^+^ in pHEL3	This study
pUWM500	*hp0231* ^+^ in pHEL2	This study
pUWM525	Km^r^ *hp0231* ^+^ in pET28a	This study

**Table 2:** Primers used in this study.

Name	Sequence 5′ - 3′	Orientation/Restriction site
**1HP231Xh**	GTGCTCGAGGCCTGCTCTTCATCAATAACTTTAG	Forward/XhoI
**2HP231**	ATCCACTTTTCAATCTATATCCAACACACTCGCTCTTAATATC	Reverse
**3HP231**	CCCAGTTTGTCGCACTGATAATTGAATCTGGCGTGATTAAGG	Forward
**4HP231**	TAGGATCCCTTGTGGGGATTTGTAGGTC	Reverse/BamHI
**Cat1**	GATATAGATTGAAAAGTGGAT	Forward
**Cat2**	TTATCAGTGCGACAAACTGGG	Reverse
**5aHP231**	AAGCGATGATGTGCAATTAG	Forward
**5bHP231**	GAGCGTTAATATCGTATTGG	Reverse
**231expI**	GAGGCCATGGCTAATGACAAACGGATGCAG	Forward/NcoI
**231expII**	GTGCTCGAGTGCCTTATAATGGTATAAGAA	Reverse/XhoI
**HP232F**	AGCGCGTTAGTGATGGTGAG	Forward
**HP232R-RT**	GCTGGTAATTAGGGTTATTG	Reverse

Restriction recognition sites introduced for cloning purposes are underlined. All primers were designed on the basis of *H. pylori* 26695 genome nucleotide sequence.

The *E. coli* strain TG1 was used as a host for the construction and preparation of recombinant plasmids. The *E. coli* strain BL21 was used to overexpress pUWM382 and pUWM525. The *E. coli* strains JCB817 and JCB818 were employed for complementation experiments of *E. coli dsbA* mutant by HP0231. *E. coli* strains were grown at 37°C on solid or liquid Luria-Bertani (LB) medium or on M63 minimal medium [20]. When needed, media were supplemented with antibiotics at the following concentrations: 100 µg ml^−1^ of ampicillin, 30 µg ml^−1^ of kanamycin and 20 µg ml^−1^ of chloramphenicol.

### General DNA manipulations

Standard DNA manipulations were carried out as described earlier [21] or according to the manufacturer's instructions (A&A Biotechnology). Polymerase chain reactions (PCR) were performed with PrimeStar HS DNA Polymerase (Takara) or HotStar HiFidelity Polymerase (Qiagen) under standard conditions. Synthetic oligonucleotides synthesis and DNA sequencing were performed by Genomed S.A.,Warsaw, Poland.

### Natural transformation of *H. pylori*

The naturally competent *H. pylori* N6 was grown on BA plates for 24 h. Subsequently, bacteria were plated onto fresh plates for 5 h. Then 0.5–1 µg of plasmid DNA was added and plates were incubated for 22 h. Afterwards bacteria were transferred onto a plate supplemented with chloramphenicol or kanamycin and transformants were grown for 5 days.

### Allelic exchange mutagenesis of *dsb* genes in *H. pylori*

To inactivate *hp0231*, the recombinant vector was constructed by a two-step PCR method [22]. Briefly, primers Cat1–Cat2, were used to amplify the *cat* gene from pRY109. The upstream and downstream regions of the *hp0231* gene were amplified from *H. pylori* 26695 genomic DNA using two pairs of primers specific for *hp0231* and its flanking regions, 1HP231Xh-2HP231 and 3HP231-4HP231, respectively. The 2HP231 and 3HP231 primers contained 5′ leader nucleotide sequences complementary to Cat1 and Cat2, respectively. Each PCR product was purified by Gel-Out extraction kit (A&A Biotechnology). Next a mixture of three purified products (in equal amounts) was used as a template in a single PCR reaction, using primers: 1HP231Xh-4HP231. Subsequently, the resulted PCR product, containing the *cat* gene inserted between two *hp0231* arms in the same transcriptional orientation as the *hp0231* gene, was purified and cloned into pGEM-T Easy generating a suicide plasmid pUWM378. Sequence analyses confirmed the correct construction of pUWM378 and the recombinant plasmid was introduced into *H. pylori* N6 by natural transformation.

A *hp0231::cat* mutant was obtained by a double crossing over by using pUWM378, and verified by PCR analysis using a pair of primers 5aHP231-5bHP231. The lack of HP0231 in the proteome of the mutated strain was confirmed by Western blot analysis using specific rabbit polyclonal anti-HP0231 antibodies.

Inactivation of *dsbI (hp0595*) was carried out by transforming a *H. pylori* N6 wild type strain with the previously constructed pUWM305 plasmid [10]. Transformants were grown for 5 days on selective plates supplemented with kanamycin. Allelic exchange in mutagenesis resulting in a *dsbI*:: *aph* mutant was confirmed by PCR. Additionally, the lack of DsbI in the proteome of the mutated strain was verified by Western blot analysis using specific rabbit polyclonal anti-DsbI antibodies.

### Construction of *hp0231* ^+^ plasmids for complementation experiments

To analyze the complementation of the *hp0231* mutation in *H. pylori* N6 and the *dsbA* mutant in *E. coli* JCB 816 by HP0231, two recombinant plasmids were constructed based on shuttle *E. coli*/*H. pylori* plasmids: pHEL3 and pHEL2. Because the *H. pylori hp0231* mutant and the *E. coli dsbA* mutant carry different genes responsible for antibiotic resistance we used two different plasmids as a starting point of these experiments. However, both plasmids had the same replication system, thus were present in similar copy number. The *hp0231* coding nucleotide sequence with its own promoter was amplified from *H. pylori* 26695 genomic DNA, using a pair of primers: 1HP0231Xh-4HP0231. The purified PCR products as well as the shuttle plasmids were digested with XhoI/BamHI and ligated together to form pUWM397 and pUWM500. Correct construction of pUWM397 and pUWM500 was confirmed by sequencing. Next, pUWM397 was introduced into *H. pylori* N6 lacking *hp0231* and pUWM500 was used for the *E. coli* DsbA complementation tests.

### Construction of *dsbI* ^+^ plasmids for complementation experiments

The recombinant plasmid pUWM336 was introduced into *H. pylori* PR305 by natural transformation (described above) in order to complement a mutation in the *dsbI* gene.

### RT-PCR

RT-PCR reactions were carried out as described earlier [23]. Total RNAs were extracted from *H. pylori* N6 and *H. pylori* PR378 using the standard TRIzol procedure (Invitrogen). After DNase I treatment, RNA was reverse-transcribed using Omniscript Reverse Transcriptase (Qiagen) and a primer HP232R-RT. PCR reactions without reverse transcriptase were used to determine whether RNA was free of DNA contamination. PCR reactions performed on cDNA were carried out with a pair of primers: HP232F-HP232R-RT.

Standard protein manipulations were carried out as described earlier [21].

### Overexpression and purification of HP0231

A HP0231 expression vector was constructed by amplifying the region encoding the mature HP0231 protein (without the signal sequence, amino acids 1–24), from the chromosome of *H. pylori* 26695 with primers 231expI and 231expII. For cloning the insert into pET22b and pET28a, NcoI and XhoI restriction enzymes were used to yield plasmids, pUWM382 and pUWM525, respectively. Both plasmids carried HP0231-His_6_ translation fusion. The periplasm-located HP0231 was overexpressed from pUWM382 by autoinduction [24] and then purified by affinity chromatography, dialyzed against PBS (Sigma) and later used for rabbit immunization (Animal Facility, Faculty of Biology, University of Warsaw). The anti-HP0231 rabbit serum was specific and recognized native HP0231, as was verified by Western blot analysis.

For the biochemical experiments the protein was expressed and purified from *E. coli* BL21 harboring pUWM525. Expression was induced with 1 mM IPTG at A_600_∼0.6. After 4 h, cultures were centrifuged and the cell pellet was suspended in 50 mM sodium phosphate, pH 8.0, 300 mM NaCl. Cells were disrupted by 2 passes through a French Press Cell at 1200 PSI. The cell lysate was centrifuged and the resulting supernatant was applied onto HisTrap column (Qiagen). The protein was eluted with an imidazole gradient. Fractions containing HP0231 were pooled and loaded onto a PD-10 column (GE Healthcare) equilibrated with 50 mM sodium phosphate, pH 8.0, 150 mM NaCl. To obtained higher purity HP0231 fractions were pooled and diluted 10-fold with 20 mM Tris, pH 8.0 and later loaded onto Sepharose column equilibrated with the same buffer. The protein was eluted with NaCl gradient. Fractions containing HP0231 were pooled, concentrated and loaded onto a PD-10 column equilibrated with 50 mM sodium phosphate, pH 8.0, 150 mM NaCl.

### *In vivo* redox state of HP0231

The redox state of HP0231 was visualized by alkylating the free cysteine residues using 4-acetamido-4′-maleimidylstilbene-2,2′-disulfonic acid (AMS, Invitrogen). This agent can only modify covalently free thiols, resulting in a molecular mass increase of 490 Da. Briefly, bacteria were harvested from BA plates after 24 or 48 h of incubation under microaerobic conditions. Samples were standardized using OD_600_ of the culture and ice-cold trichloroacetic acid (TCA, final concentration 10% v/v) was immediately added directly to the culture. Whole-cell proteins were precipitated and collected by centrifugation, then washed with ice-cold acetone and dissolved in 50 mM Tris-HCl (pH 7.5), 10 mM EDTA, 0.1% SDS containing 20 mM AMS by agitation for 45 min at 37°C. The proteins in non-reducing Laemmli buffer were resolved by 18% SDS-PAGE without reducing agent. HP0231 was then detected by immunoblot analysis using an anti-HP0231 antibody.

As controls, we used samples previously treated with 100 mM DTT for 30 min at room temperature before precipitation of the proteins with TCA.

### Insulin reduction assay

The ability of HP0231 and EcDsbA to catalyze the reduction of insulin in the presence of DTT was determined as previously described [25].

### Determination of the redox potential of HP0231

The redox potential of HP0231 was fluorimetrically determined at pH 7 at room temperature from the equilibrium constant with glutathione as previously described [25]. Briefly, experiments were performed at room temperature in a buffer containing 100 mM sodium phosphate (pH 7.0) and 1 mM EDTA. HP0231 (1 µM) was incubated for 12 h in the presence of 0.1 mM GSSG and varying concentrations of GSH (0.4 µM to 8 mM). For different GSH/GSSG ratios, the fraction of oxidized and reduced HP0231 was determined from the increase of the tryptophan fluorescence upon reduction. The fraction (R) of each of the reduced proteins at equilibrium with glutathione was measured using specific fluorescence of the protein at 330 nm (excitation 295 nm). The equilibrium constant (K_eq_) was determined by fitting the obtained data to the equation:
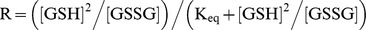
The redox potential was calculated using the Nernst equation. The value of −240 mV was used as a standard potential for glutathione.
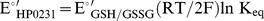



### DTT sensitivity assay

DTT sensitivity experiments were performed as previously described [26] with some modifications concerning *H. pylori* growth requirements. Briefly, a freshly prepared 1 M DTT (Sigma) stock solution was dissolved in molten BA supplemented with 10% horse blood to the final concentration of 8 mM DTT (for *H. pylori* assay) or in molten LB agar to the final concentration of 12 mM DTT (for *E. coli* assay). The amount of DTT used completely inhibits the growth of the *E. coli* strain JCB817, but not the wild type parental strain. The DTT agar plates were used within 30 min of pouring to prevent oxidation of DTT by air. Exponentially growing cultures were serially diluted 10-fold, and 7 µl aliquots were spotted on the plates. The growth was observed after 48–72 h of incubation for the *H. pylori* assay and after overnight incubation at 37°C for the *E. coli* assay. The experiments were conducted in triplicate.

### Motility assays

*H. pylori* strains were grown in BHI supplemented with 10% heat-inactivated FCS until mid-log phase. Next, cells were inoculated on Mueller-Hinton (MH) soft agar plates containing 0.35% (w/v) agar and 10% (v/v) FCS with a sterile toothpick and incubated for 4 to 5 days at 37°C under microaerobic conditions.

*E. coli* cells were grown in liquid culture in LB broth until the OD_600_ value was close to 1. Then bacteria were inoculated on LB soft agar plates containing 0.35% (w/v) agar with a sterile toothpick and incubated for 18 h at 30°C.

### Transmission electron microscopy

Bacteria were grown on BA plates for 48 h under microaerobic conditions, then washed three times with PBS and suspended in 1.5% (v/v) glutaraldehyde. Subsequently, the bacteria were transferred to a 400-mesh carbon – coated copper grid by floating the grid, coated side down on a drop of *H. pylori* culture fluid for 30 s. Next, the bacteria were negatively stained by floating the grid on a drop of 1% (w/v) phosphotungstic acid solution for 20 s. The excess of reagent was washed twice by floating the grid on a drop of water for 10 s. The grid was allowed to dry in air. Samples were examined on an electron microscope JEM 1400 (JEOL Co., Japan, 2008).

Measurements of 100 cells/strain were taken manually from TEM images by drawing the central axis of each cell running from pole to pole, in the iTEM 5.0 (Build 1235) Olympus Soft Imaging Solution GmbH).

### Alkaline phosphatase (AP) assay

AP activity was measured as previously described [16] with some modifications. Briefly, *E. coli* cell cultures were grown at 37°C in minimal medium M63 until they reached mid-log phase. Then, the cells were pelleted by centrifugation, washed twice with Tris-HCl (pH 8.0), and resuspended in Tris-HCl (pH 8.0). The OD_600_ was measured spectrophotometrically. Samples (1 ml) were equilibrated for 5 min in water bath at 28°C, then 200 µl *p*-nitrophenol phosphate [0.4% (w/v) in Tris-HCl (pH 8.0)] was added, and time was recorded. The reaction was allowed to proceed at 28°C until the development of yellow color was observed. At this point the reaction was stopped by adding 200 µl 1 M KH_2_PO_4_. Samples were centrifuged before the *A*_420_ and *A*_550_ measurements.

### Copper sensitivity assay

For copper sensitivity assay bacteria were grown in BHI media supplemented with 8 mM or 10 mM CuCl_2_. Strains were grown at 34°C.

### Statistical analysis

Statistical analyses of variance (ANOVA) and ANOVA specifying the “Welch” option controls for unequal variances followed by the Tukey's post hoc test, as well as the mean and SD calculations were carried out using MS Excel 2007 with SigmaXL.

## Results

### 1. Determination of the *in vivo* redox state of HP0231

When we started this work, we first performed fold-recognition analyses that suggested that the fold of HP0231 resembles that of EcDsbG. This was confirmed by the recently published structure of HP0231 [27]. In this structure, HP0231 appears as a V-shaped dimeric protein, which is reminiscent of the oxidoreductases that function in the reductive pathways present in the *E. coli* periplasm. However, both the facts that the *H. pylori* genome does not encode a classical monomeric DsbA protein, despite the presence of a *dsbB*-like gene (*hpdsbI*), and that the catalytic site of HP0231 is identical to that of EcDsbA (both proteins have a CPHC active site) lead us to suspect that HP0231 might be involved in disulfide bond formation rather than in disulfide isomerization.

Thus, to clearly define whether HP0231 functions in the oxidizing or the reducing pathway, we first decided to determine the redox state of that protein *in vivo*. The *in vivo* redox state of an oxidoreductase usually reflects its activity in the cell, i.e., proteins that function in the oxidizing pathway, such as EcDsbA, are maintained oxidized *in vivo*, whereas proteins that function in the reducing pathway are maintained predominantly reduced.

**Figure 1:**
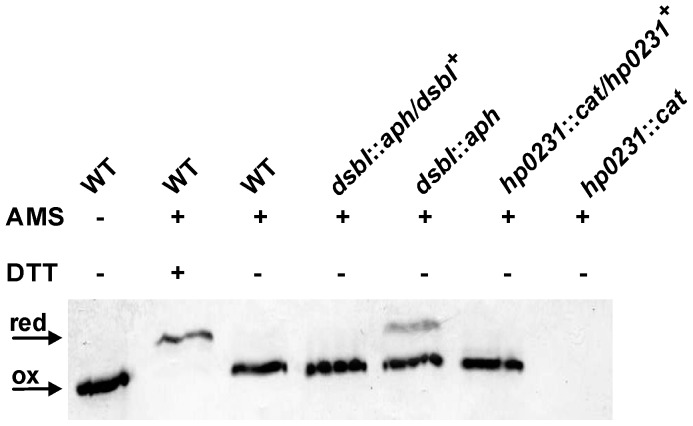
Redox state of HP0231 in WT, mutants: *dsbI*::*aph, hp0231*::*cat* and complemented strains. Bacterial cultures were treated with 10% TCA, followed by alkylation with AMS. Cellular proteins including the reduced (red; DTT treated, modified with AMS) and the oxidized (ox; non-modified with AMS) controls were separated by 18% SDS-PAGE under non-reducing conditions, and Western blot analysis using antibodies against HP0231 was performed. Each lane contains proteins isolated from the same amount of bacteria.

We used the AMS-trapping technique to determine the *in vivo* redox state of HP0231. We found that HP0231 is present in the oxidized form in wild-type cells, which suggests that HP0231 functions in the oxidizing pathway in *H. pylori*. We then tested whether deletion of *hpdsbI* affects the redox state of HP0231 (remembering that EcDsbB is the protein that recycles EcDsbA). As shown in Figure 1, the deletion of the *hpdsbI* results in the accumulation of a noticeable amount of HP0231 in the reduced state. Thus, these data strongly suggest that HP0231, despite its structural resemblance to EcDsbG, functions in the oxidizing pathway in the *H. pylori* periplasm and that HP0231 is maintained oxidized by HpDsbI.

### 2. Phenotypic characterization of HP0231 mutated cells

To shed more light on HP0231's function, we constructed an isogenic *hp0231* knock-out strain by allelic exchange methodology. A recombinant plasmid (based on a vector non-replicating in *Helicobacter* cells) was used for the mutagenesis. It contained the *hp0231 gene* disrupted by insertion of a chloramphenicol resistance cassette into the gene coding sequence. The correctness of the plasmid construction was confirmed by PCR and DNA sequencing. The expected disruption of the chromosomal *hp0231* locus as a result of the double cross-over recombination event was verified by PCR using appropriate pairs of primers. The loss of the wild-type *hp0231* gene product was also confirmed by Western blotting of whole-cell proteins with specific anti-HP0231 antibodies (Figure S1). We also verified that the antibiotic cassette disrupting the *hp0231* gene (introduced in the same transcriptional orientation as the mutated gene) causes no polar effect on the expression of the adjacent gene (*hp0232*) using RT-PCR (Figure S2).

**Figure 2:**
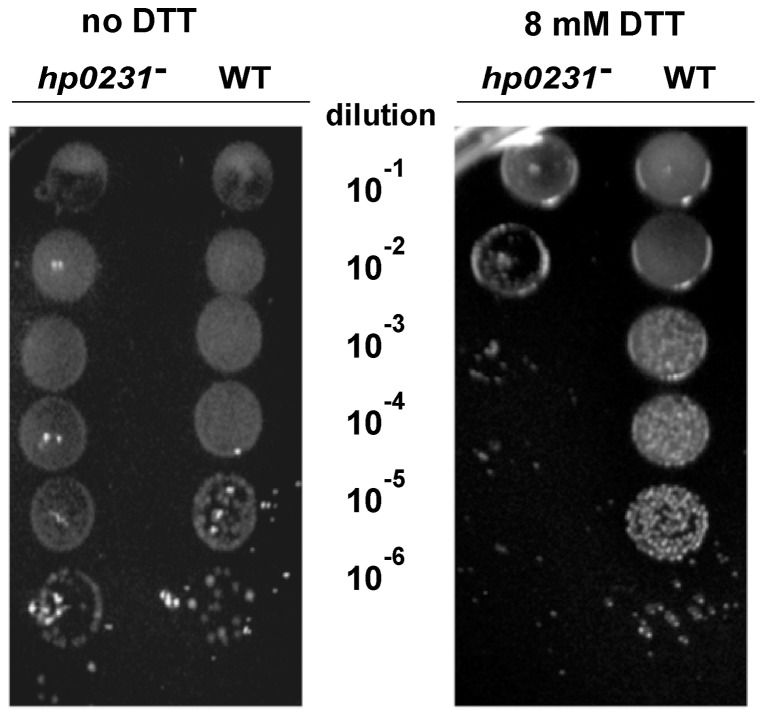
The *H. pylori* N6 *hp0231*::*cat* mutant is DTT sensitive. Exponentially growing *H. pylori* wt and *hp0231*::*cat* cultures were ten-fold serial diluted and spotted on BA plates without (panel A) or with (panel B) 8 mM DTT, and incubated at 37°C. The mutant shows reduced growth after 3 days of incubation on plates containing DTT.

As the *E. coli dsbA* mutant is sensitive to dithiotreitol (DTT), we first decided to compare the DTT sensitivity of the *hp0231* mutant to that of wild type cells. We found that *H. pylori* lacking HP0231 is DTT-sensitive and shows reduced growth compared to the wild-type parental strain (Figure 2).

**Figure 3:**
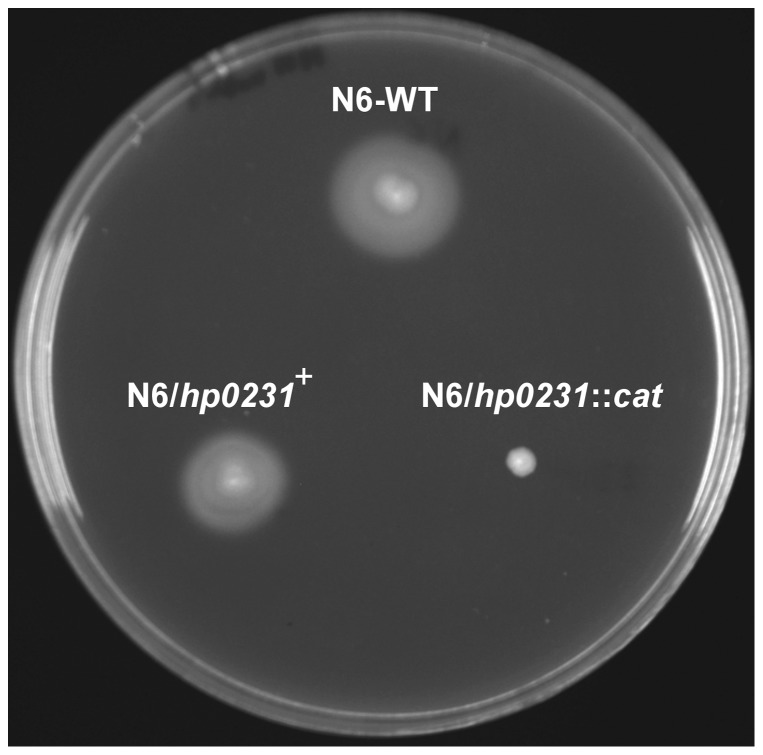
Motility of *H. pylori* N6 strains: wt, *hp0231::cat* and *hp0231::cat* complemented *in trans* by puWM397 (*hp0231^+^*). Bacterial motility was monitored after 4 days of incubation on 0.3% MH-agar plates containing 10% FCS. The *hp0231* mutant strain is non-motile.

**Figure 4:**
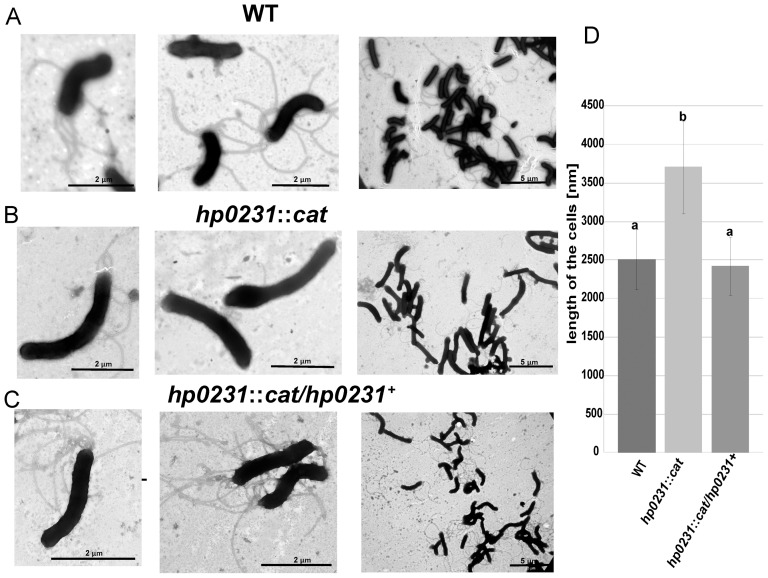
Comparison of *H. pylori* N6 cells (wt, *hp0231::cat* and *hp0231::cat* complemented *in trans* by puWM397 (pHel2/*hp0231*) morphology by transmission electron microscopy (TEM). A) WT, B) *hp0231*::*cat* and C) *hp0231*::*cat/hp0231^+^* (*hp0231*::*cat* complemented *in trans* by wild type copy of *hp0231*). D) The diagram illustrates mean lengths and standard deviations of 100 bacterial cells in nm. Error bars with the different letters indicate a significant difference (p<0.01) in length between the *H. pylori* N6 wt cells and the mutant strain, and the mutant and the complementant strain (Welch ANOVA followed by post hoc Tukey's test).

Bacteria from many genera that contain a mutation in *dsbA* or *dsbB* are non-motile, because of their inability to produce functional flagella [28]. As shown in Figure 3, the *hp0231* mutant is non-motile. To clarify the reason for the lack of motility, we performed transmission electron microscopy experiments to visualize potential changes in the *H. pylori* flagellar elements and/or in cellular morphology. As shown in Figure 4, we did not observe any defect in the flagella distribution or structure. We observed, however, some changes in the mutant morphology. The *hp0231* gene inactivation yields cells either with straight-rod or curved-rod morphology. Additionally, on average, these cells are longer (almost 50% longer) than the wild-type cells. A wild-type phenotype could be restored by *hp0231* complementation *in trans*.

### 3. HP0231 complements an *E. coli dsbA* mutant

**Figure 5:**
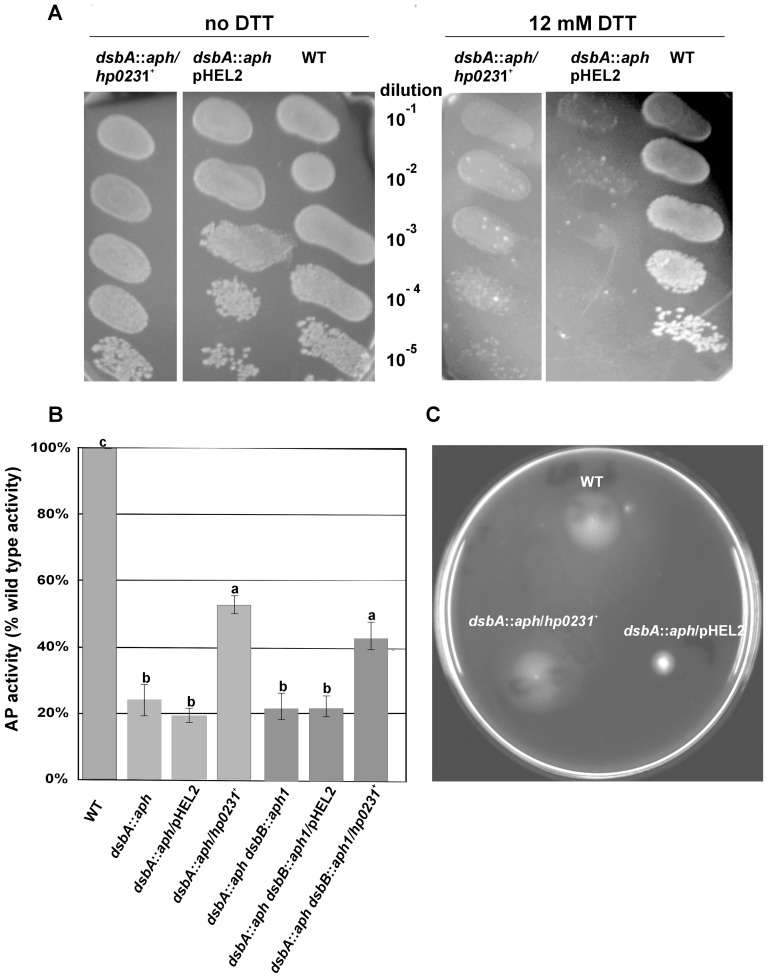
HP0231 restores the *E. coli dsbA* ^−^ wild type phenotype in three independent functional assays. As a negative control *E. coli dsbA*::*aph* was transformed with an empty pHEL2 vector. **Panel A** DTT sensitivity assay; **panel B** alkaline phosphatase assay. The bars represent average activity of three independent experiments with wild type set to 100% activity; error bars with the different letters indicate a significant difference (p<0.01) in relative alkaline phosphatase activity between the *H. pylori* N6 wt cells and the mutant strains, and the complementant strains (in ANOVA followed by post hoc Tukey's test). Alkaline phosphatase activity of wild type and *dsb* mutants and complementants strains was performed in M63 minimal media; **Panel C** motility assay.

We then tested the ability of HP0231 to complement the *E. coli dsbA* mutant. *E. coli* strains lacking a functional *dsbA* exhibits a pleiotropic phenotype such as loss of motility, DTT sensitivity and low alkaline phosphatase activity [4]. HP0231 was expressed in the *E. coli dsbA* mutant to determine the ability of this protein to suppress the defects associated with the lack of disulfide bond formation. Expression of the protein was confirmed by Western blot using a specific rabbit anti-HP0231 antibody (data not shown). We first tested whether the expression of HP0231 in the *dsbA* mutant leads to increased levels of alkaline phosphatase activity and found that expression of HP0231 restores about 50% of the PhoA activity measured in the wild-type strain (Figure 5B). Then, in order to assess whether the activity of HP0231 in *E. coli* is dependent on the presence of *E. coli* DsbB, we introduced a plasmid-encoding HP0231 into *E. coli* JCB818 cells (*dsbA dsbB* double mutant). However, we observed that the absence of EcDsbB only slightly decreases the alkaline phosphatase activity.

We also found that the expression of HP0231 in the *E. coli dsbA* mutant both restores the motility of this strain (Figure 5C) and suppresses the DTT-sensitivity (Figure 5A).

Taken together, our results clearly indicate that HP0231, despite its structural resemblance to EcDsbG, can complement the *E. coli dsbA* mutant, suggesting that HP0231 is able to catalyze disulfide bond formation in the bacterial periplasm.

### 4. HP0231 does not complement an *E. coli dsbC* mutant

We also tested whether the expression of HP0231 in the *dsbC* mutant restores viability characteristic for wild-type cells under copper stress conditions. We found that HP0231 does not complement the *dsbC* mutation (Figure S3) suggesting that HP0231 is not active in the isomerization pathway.

### 5. Biochemical characterization of HP0231

**Figure 6:**
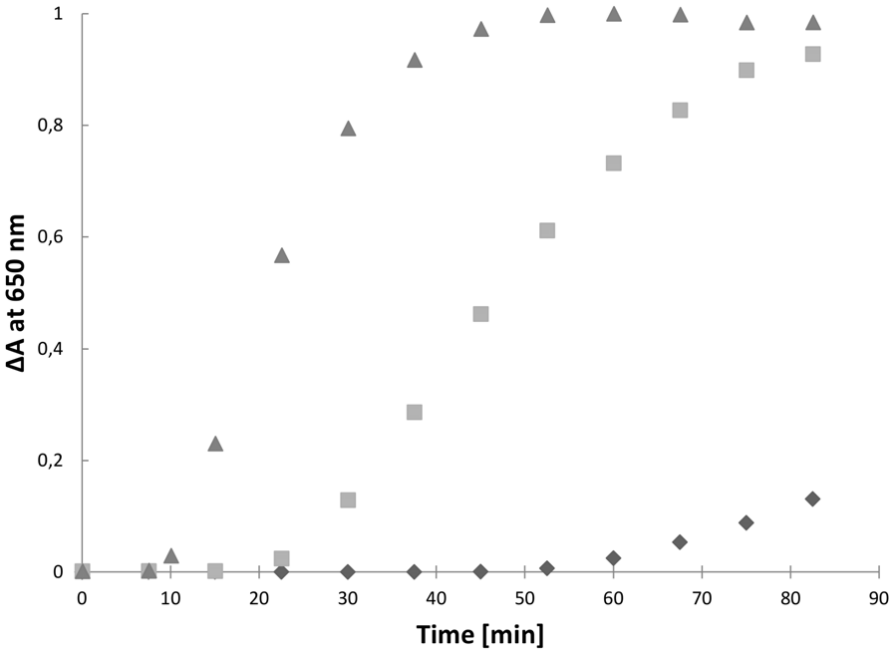
The insulin reduction assay. The reaction mixture contained 150 µM insulin in potassium phosphate buffer, pH 7.0 and 2 mM EDTA. The reaction was performed in the absence (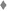) or presence of 10 µM EcDsbA (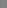), 10 µM HP0231 (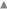). Reactions started by adding DTT to the final concentration of 1 mM. The changes in the absorbance at 650 nm as a function of time were measured. Three independent experiments were performed.

To characterize the biochemical properties of HP0231, we produced a recombinant protein in *E. coli*. We first determined whether HP0231 is able to catalyze the reduction of insulin by DTT. Insulin contains two intramolecular disulfide bonds that connect the A and B chains: reduction of these disulfide bonds causes the precipitation of the B chain, which can be monitored by following the increase of turbidity at 650 nm [29], [30]. The insulin reduction assay is commonly used to determine whether a protein can function as an oxidoreductase, regardless of its function in the reducing or the oxidizing pathway *in vivo*. As shown in Figure 6, HP0231 catalyzes insulin reduction, even more efficiently than EcDsbA.

**Figure 7:**
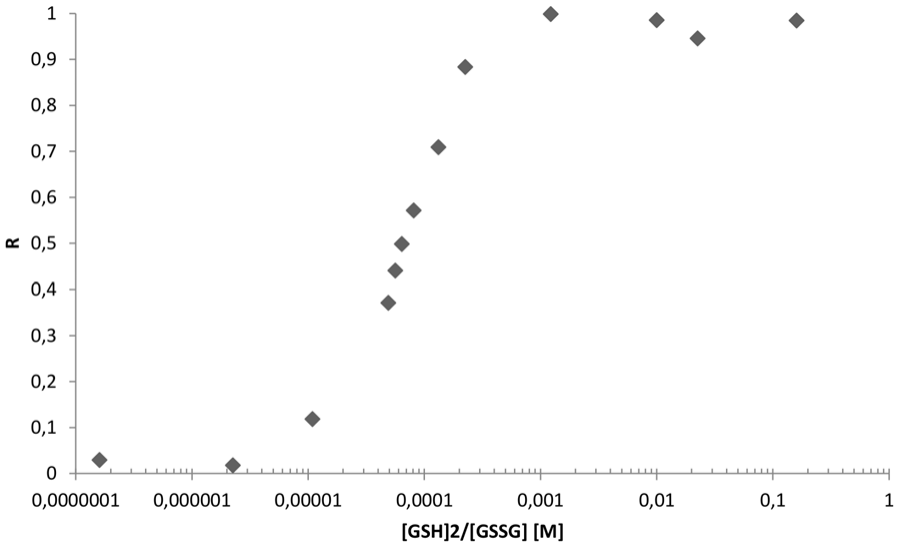
Redox equilibrium of *H. pylori* HP0231 with glutathione. The fraction of reduced (R) HP0231 was determined using the specific HP0231 fluorescence at 324 nm.

We also determined the redox potential of HP0231 by equilibrating this protein in a series of redox buffers containing various ratios of reduced and oxidized glutathione (Figure 7). The standard redox potential at 20°C calculated for HP0231 is −116 mV, which is similar to the redox potential of EcDsbA (−120 mV) [31], [32].

## Discussion

The *E. coli* Dsb system is considered to be the paradigm of oxidative protein folding pathways in bacteria. However, bioinformatic analyses of whole-genome sequencing data have revealed that many bacterial disulfide bond-forming systems diverge from the *E. coli* machinery. It is therefore important to characterize in details the oxidative folding pathways operating in bacteria other than *E. coli*, including pathogens such as *H. pylori*. As many virulence factors are indeed stabilized by the formation of one or more disulfide bonds, the characterization of *H. pylori* Dsb proteins may facilitate a future discovery of an effective anti-*Helicobacter* drug. A multiparameter selection of *H. pylori* antigens has also marked out HP0231 as a potentially highly protective antigen for vaccine development. Mouse immunization with purified HP0231 indicated its high protective efficacy [33], [34]. Immunization of human volunteers with *Salmonella* Ty21a, the licensed typhoid fever vaccine, producing HP0231 indicated an antigen-specific CD4^+^ T cell response and mild protective effect [35]. The bioinformatic analysis of *H. pylori* (strain 26695) proteome, conducted with the general localization predictor PSORTb3 [36] and with the meta-predictor MetaLocGramN specialized in predicting subcellular localization for proteins from Gram-negative bacteria [37], indicated that this bacterium has around 150 non-cytoplasmic proteins, containing at least two cysteine residues. Thus, these proteins are potential targets of the *H. pylori* Dsb system (J.M. Bujnicki and M. Magnus, International Institute of Molecular and Cell Biology in Warsaw, personal communication). The list of proteins that potentially form disulfide bonds includes virulence factors such as the vacuolating cytotoxin VacA or proteins that are components of the type IV secretion apparatus encoded by genes clustered in the Cag pathogenicity island. Effectors molecules delivered by this secretion system into eukaryotic cells are involved in interactions with many of the host signal-transduction pathways. Previously, we also demonstrated that a *H. pylori* mutant impaired in disulfide bond formation exhibits a greatly reduced ability to colonize mice gastric mucosa [9].

Here, we report the functional and biochemical characterization of HP0231, a *H. pylori* protein from the Dsb family. First, we show that HP0231 is maintained oxidized in *vivo*, similarly to EcDsbA. Our data suggest that the protein that maintains HP0231 oxidized is HpDsbI. We also characterized the phenotype of a *hp0231* mutant strain and found that it is reminiscent of the phenotype of *E. coli dsbA* mutants. Whereas an *E. coli dsbA* ^−^ strain exhibits a number of phenotypes, no consistent phenotype has yet been found for the *E. coli dsbG* ^−^ strain. *E. coli* lacking the periplasmic disulfide isomerase *dsbC* are sensitive to the redox-active metal copper as DsbC is required for rearrangements of copper-catalyzed non-native disulfides [1], [20]. Expression of the HP0231 in the *E. coli dsbA* mutant complements the motility defect and the sensitivity to DTT, and partially restores the ability to oxidatively fold alkaline phosphatase. However, expression of HP0231 does not complement the *E. coli dsbC* mutant. Measuring the redox potential of the protein revealed that it has a high redox potential similarly to *E. coli* DsbA. Additionally HP0231 is able to catalyze reduction of insulin in the presence of DTT whereas EcDsbG is not active in this assay [2], [38].

Altogether, our results indicate that HP0231 functions in the oxidizing pathway involving HpDsbI, and that HP0231 is the functional equivalent of EcDsbA. For that reason, we propose to call this protein HpDsbA.

The structure of HP0231 has been recently solved. Intriguingly, HpDsbA appears as a V-shape protein, like EcDsbC and EcDsbG, two proteins functioning in reducing pathways. Because of this structural similarity, HP0231 was proposed to be the functional homologue of EcDsbG in *H. pylori*. Moreover, HP0231 was shown *in vitro* to be able to reduce HP0518, which is a putative L,D-transpeptidase presenting a single catalytic cysteine residue [27], [39].

These latter data require further clarification as HP0518, according to Asakura *et al.*, exerts its activity in the cell cytoplasm [39]. However, HP0518 has a classical signal sequence and it cannot be excluded that under certain conditions HP0518 maybe secreted [39]. As explained above, our data, and particularly the fact that HP0231 is maintained in an oxidized state *in vivo*, provide strong arguments against the involvement of this protein in the reducing pathway.

Although individual domains of HP0231 display structural resemblance to the corresponding domains of EcDsbG, there are significant structural variations between the two proteins, which result in different dimer structures. In HP0231, the linker helix is slightly longer as compared to EcDsbG resulting in a different orientation of the C-terminal catalytic domain with respect to the N-terminal dimerization domain [27]. There are also major differences in the amino acid residues lining the V-shaped cleft of HP0231 and EcDsbG. The inner surface of the *H. pylori* HP0231 cleft is lined with positive residues, which are absent in EcDsbG.

Both the flagellum and the helical shape allow *H. pylori* to penetrate the mucosa and then to colonize and later persist in the host stomach [40]. However, the mechanisms by which *H. pylori* control its helical shape and cellular motility are not completely understood. We showed that the *hp0231* inactivation results in cell-shape changes and lack of motility, which for now cannot be explained as the cells are normally flagellated. Similarly, *Campylobacter jejuni* mutants with a defective *pflA* (*paralyzed flagellar protein*) gene are also non-motile but possess a flagellum [41], [42]. *H. pylori* HP1274, homolog of PflA, is a predicted extracytoplasmic protein with six cysteine residues. Thus, this protein can be a potential target of HP0231. However this hypothesis requires further clarification.

Additional work will also be needed to understand the role that the Dsb system plays in cell morphology. Remarkably, the shape of *H. pylori* cells lacking HP0231 resembles that of the *H. pylori ccmA* (curved cell morphology) mutated strain described by Sycuro et al. [43] and that of the *ccrp* mutant (HP0059 – one of coiled coil rich proteins) described by Waidner et al. [40].

In conclusion, this study provides new insight into the *H. pylori* disulfide bond formation system, and led to the characterization of the first dimeric oxidoreductase functioning in the oxidizing pathway.

## Supporting Information

Figure S1 **Confirmation of** ***hp0231*** **mutation by Western-blot analysis.** *H. pylori* wt N6 and N6 *hp0231*::*cat* proteins (the whole cell lysate) were separated by 12% SDS-PAGE and electrotransfered onto a nitrocellulose membrane. Specific rabbit serum with antibodies against HP0231 were used to verify the lack of HP0231 in N6 *hp0231*::*cat* mutant cells. The asterisks denote unknown proteins recognized by the antiserum. The relative positions of the molecular weight markers (lanes M) are listed on the left (in kilodaltons).
(TIF)

Figure S2 **RT-PCR analysis of** ***H. pylori hp0232*** **transcription from the wild-type or** ***hp0231*** **::** ***cat*** **mutant chromosomal DNA.** Equal amounts of mRNAs isolated from *H. pylori* cells (wt – lane 1 and *hp0231*::*cat* mutant – lane 3) were reverse-transcribed using primer hp232R and the resulting cDNA was PCR-amplified with a pair of primers, hp232F and hp232R. To the control reactions (lanes 2 & 4) reverse transcriptase was not added. The relative positions of the DNA molecular length markers (lanes M) are listed on the left.
(TIF)

Figure S3 **HP0231 does not restore the** ***E. coli dsbC*** **^−^ wild type phenotype in the copper sensitive assays.** As a negative control *E. coli dsbC*::*aph* was transformed with an empty pHEL2 vector. The numbers indicate: 1 – WT, 2 - *dsbC*::*kan*, 3 *– dsbC*::*kan/hp0231^+^*, 4 *– dsbC*::*kan/hp0377*, 5 *– dsbC*::*kan/*pHEL2.
(TIF)
