# Correction: A Novel Insight into the Oxidoreductase Activity of *Helicobacter pylori* HP0231 Protein

**DOI:** 10.1371/annotation/a753231a-64d0-4038-aa1e-1a31d68f70fb

**Published:** 2012-10-04

**Authors:** Paula Roszczenko, Katarzyna A. Radomska, Ewa Wywial, Jean-Francois Collet, Elzbieta K. Jagusztyn-Krynicka

The following information was omitted from the Funding section:

This work was funded by the grant of Polish Ministry of Science and Higher Education N N303 550439 and by intramural grant of University of Warsaw 501/86- 100036. The research for this paper was in part supported by the EU through the European Social Fund, contract number UDA-POKL.04.01.01-00-072/09-00. PR obtained short term fellowship in JFC lab from EMBO no. ASTF 450.00-2010. 

