# Supplementary figures and images for: A Novel Insight into the Oxidoreductase Activity of Helicobacter pylori HP0231 Protein
Source: PLoS One. 2012 Oct 3;7(10):e46563. doi: 10.1371/journal.pone.0046563 (PMC3463561; doi:10.1371/journal.pone.0046563)

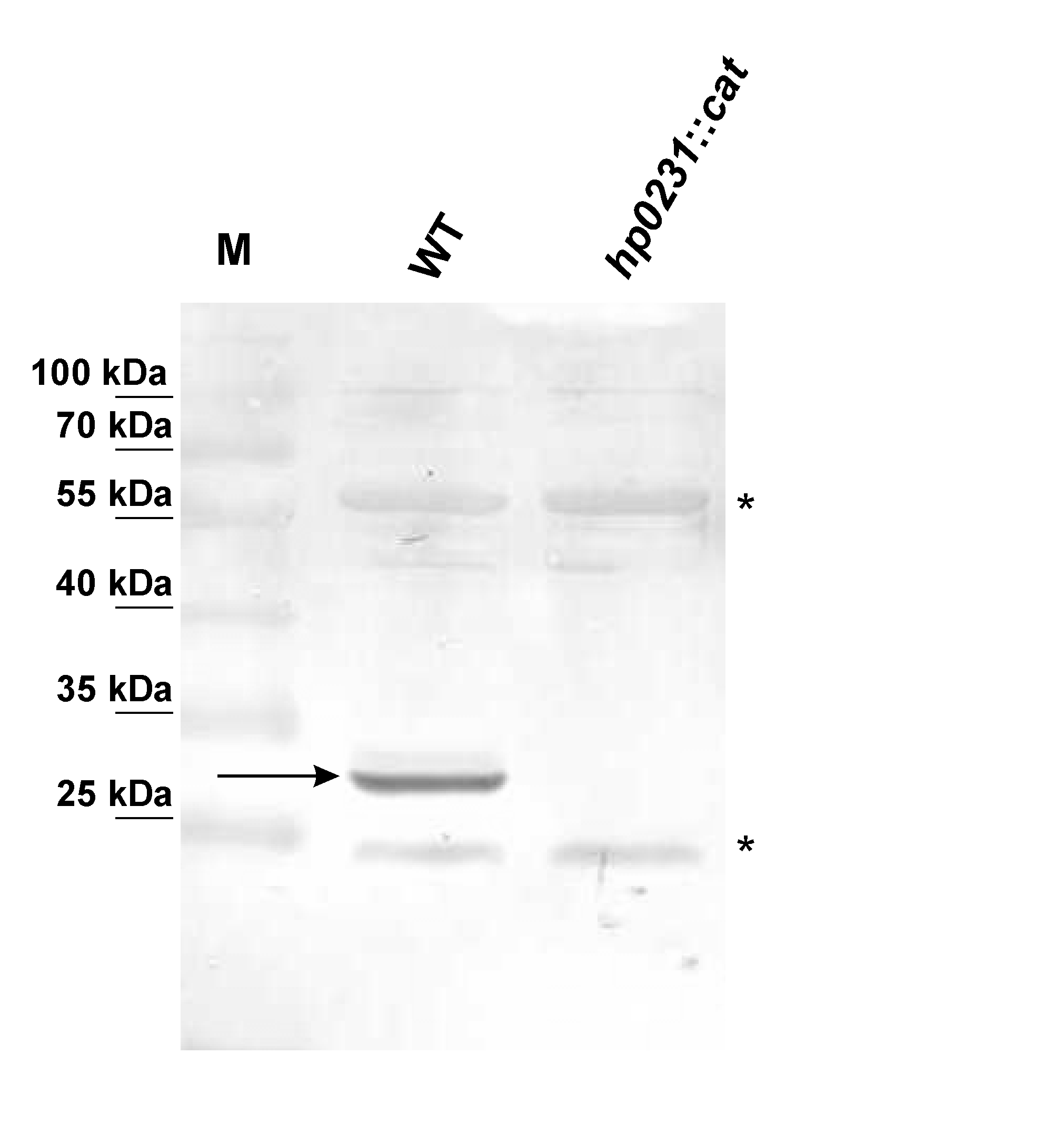

Supplement: Figure S1 — Confirmation of hp0231 mutation by Western-blot analysis. H. pylori wt N6 and N6 hp0231::cat proteins (the whole cell lysate) were separated by 12% SDS-PAGE and electrotransfered onto a nitrocellulose membrane. Specific rabbit serum with antibodies against HP0231 were used to verify the lack of HP0231 in N6 hp0231::cat mutant cells. The asterisks denote unknown proteins recognized by the antiserum. The relative positions of the molecular weight markers (lanes M) are listed on the left (in kilodaltons). (TIF) [file pone.0046563.s001.tif]

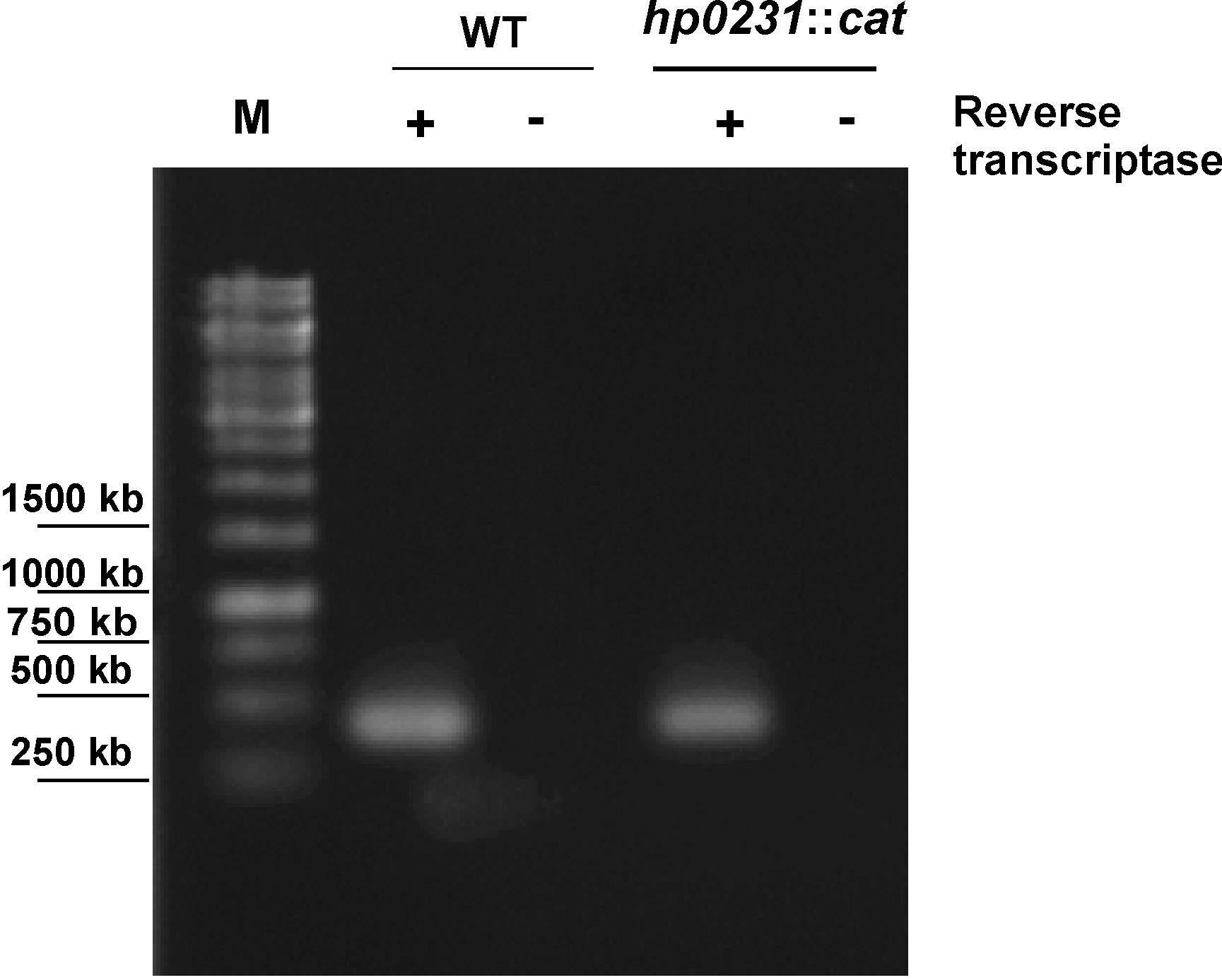

Supplement: Figure S2 — RT-PCR analysis of H. pylori hp0232 transcription from the wild-type or hp0231 :: cat mutant chromosomal DNA. Equal amounts of mRNAs isolated from H. pylori cells (wt – lane 1 and hp0231::cat mutant – lane 3) were reverse-transcribed using primer hp232R and the resulting cDNA was PCR-amplified with a pair of primers, hp232F and hp232R. To the control reactions (lanes 2 & 4) reverse transcriptase was not added. The relative positions of the DNA molecular length markers (lanes M) are listed on the left. (TIF) [file pone.0046563.s002.tif]

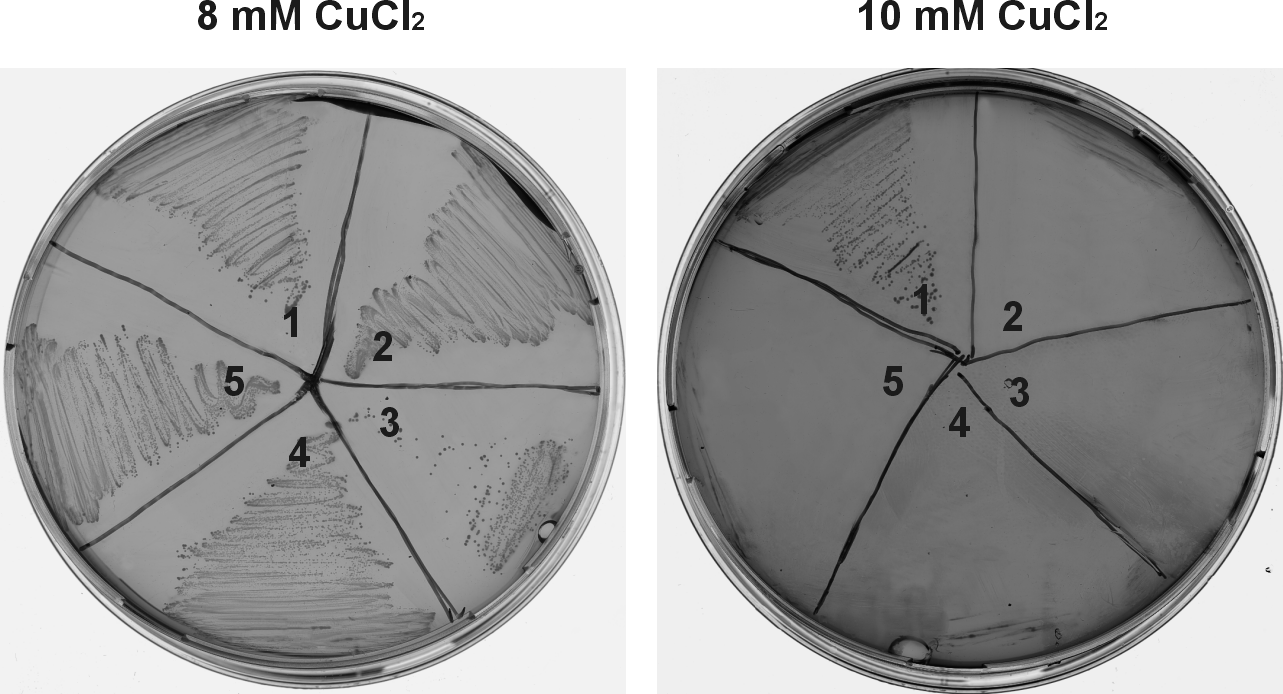

Supplement: Figure S3 — HP0231 does not restore the E. coli dsbC − wild type phenotype in the copper sensitive assays. As a negative control E. coli dsbC::aph was transformed with an empty pHEL2 vector. The numbers indicate: 1 – WT, 2 - dsbC::kan, 3 – dsbC::kan/hp0231+, 4 – dsbC::kan/hp0377, 5 – dsbC::kan/pHEL2. (TIF) [file pone.0046563.s003.tif]
